# Disease burden, influencing factors and trend-based surveillance of needlestick injuries among healthcare workers: a seven-year longitudinal study and intervention evaluation at a tertiary hospital

**DOI:** 10.3389/fpubh.2026.1833892

**Published:** 2026-06-11

**Authors:** Xiaoyu Zhu, Yue Gao, Jinfen Zhu, Fang Wu, Chunmei Shen

**Affiliations:** 1 Department of Hospital Infection Management, Shanghai Fifth People's Hospital, Fudan University, Shanghai, China; 2 Health Economic Research Institute, School of Pharmaceutical Sciences, Sun Yat-Sen University, Guangzhou, China

**Keywords:** cost of illness, COVID-19, health personnel, infusion center, linear model, needlestick injuries, public health surveillance, safety intervention

## Abstract

**Background:**

Needlestick injuries (NSIs) remain a leading occupational hazard among healthcare workers (HCWs), carrying substantial health and economic burdens. High-throughput procedural settings are particularly vulnerable, yet evidence on setting-specific interventions' effects and durability under healthcare system volatility remains limited. Existing surveillance systems for NSIs struggle to distinguish genuine risk escalation from clinical workload fluctuations. This study aims to characterize the epidemiological and economic burden of NSIs among HCWs, evaluate the effectiveness of safety interventions in the Infusion Center during post-pandemic clinical resurgence, and develop a dynamic NSIs surveillance system.

**Methods:**

A 7-year retrospective longitudinal study was conducted at a tertiary hospital across three phases: pre-, intra- and post-COVID-19 pandemic phases (P1, P2, and P3). The Infusion Center, which implemented a multi-component safety intervention bundle, served as the intervention group, with 38 other clinical departments as controls. NSIs incidence was modeled using a generalized linear mixed model (GLMM) with Poisson distribution, incorporating a department-level random intercept, a staff-size offset and hospital-wide clinical volume as a time-varying covariate. A dynamic early warning system was constructed using GLMM-derived, volume-adjusted upper control limits.

**Results:**

The Infusion Center exhibited a 22.43-fold higher NSIs risk than controls in P1 (IRR = 22.43, *p* < 0.001). Controls showed a 43% reduction in P2 (IRR = 0.57, *p* = 0.033) followed by a rebound in P3 (IRR = 1.06, *p* = 0.753). Conversely, the Infusion Center showed a sustained 66% reduction in P3 relative to P1 (IRR = 0.34, *p* = 0.0006), significantly diverging from the hospital-wide rebound trajectory (interaction IRR = 0.32, *p* = 0.005). The dynamic early warning system identified 25 alarms across controls during P3, while the Infusion Center triggered zero alarms. The intervention bundle was associated with 20 fewer NSIs cases in P3, yielding a direct medical cost avoidance of 12,776.68 RMB.

**Conclusion:**

Systematic behavior-based interventions combining leadership accountability, competency training and continuous reinforcement are associated with a sustained reduction in NSIs risk that diverged from the post-pandemic rebound observed across control departments. The dynamic early warning system represents a proof-of-concept approach to volume-adjusted occupational safety surveillance, enabling targeted resource allocation to high-risk departments.

## 1 Introduction

Needlestick injuries (NSIs) represent one of the most prevalent and serious occupational hazards among healthcare workers (HCWs) worldwide. Globally, HCWs suffered more than 2 million occupational NSIs annually (1), with the World Health Organization (WHO) estimating approximately 35 million NSIs occurring among HCWs annually on a global scale (2). The worldwide pooled prevalence of NSIs among HCWs during career time and previous 1 year was 56.2% (95% CI: 47.1–64.9) and 32.4% (95% CI: 22.0–44.8), respectively (3). In China alone, some scholars estimate there could be as many as 3.8 million NSIs among HCWs annually, a figure 10 times greater than estimates for the United States (2).

Beyond the direct transmission risk of bloodborne pathogens, NSIs impose significant psychological and economic burdens on affected HCWs. The US CDC cited estimates of the direct costs associated with initial follow-up and treatment of HCWs who sustained a NSI ranging from US$71 to US$5,000, depending on the treatment (4). These costs include laboratory testing, post-exposure prophylaxis (PEP), follow-up visits and potential productivity losses, creating substantial financial strain on healthcare systems globally.

Injection facilities and phlebotomy centers have emerged as particularly high-risk environments due to the convergence of multiple risk factors: high patient throughput creating time pressure, procedure-intensive workflows requiring frequent percutaneous interventions (5) and patient populations (oncology, geriatric and chronically ill) with difficult venous access necessitating multiple insertion attempts (6, 7). These structural vulnerabilities underscore the need for setting-specific risk stratification and targeted interventions rather than uniform hospital-wide approaches.

The COVID-19 pandemic created an unprecedented natural experiment in occupational safety, offering unique insights into NSIs epidemiology under conditions of dramatic healthcare system volatility. During pandemic lockdowns, multiple surveillance systems documented temporary NSIs rate fluctuations. Stojic et al. (8) reported that while total monthly NSIs remained relatively stable during pandemic, the rate per 1,000 hospital activities and per 1,000 hospitalized patients increased significantly mainly because HCWs were continuously equipped with personal protective equipment (PPE). Conversely, Diktas et al. (9) documented NSIs rate in Turkish hospitals declining from 27.65% (2019) to 21.4% (2020) during lockdown periods as a result of well-designed training and awareness programs.

However, these studies suffered from a critical limitation: insufficient post-pandemic follow-up to assess durability of behavioral changes. To date, few studies have investigated NSIs rate trends following pandemic with clinical volume rebound and adjustments in infection prevention and control (IPC) measures. The temporal volatility in NSIs rate raises fundamental questions about sustainability of behavior-based interventions in the absence of ongoing external pressure and embedded organizational accountability structures, which collectively underscore two critical gaps: the inadequacy of passive, knowledge-based prevention strategies to achieve durable behavioral changes and the absence of dynamic surveillance tools capable of distinguishing genuine risk escalation from fluctuations in clinical workload.

More recently, microlearning, a brief focused learning module (typically < 15 min) delivering just-in-time via digital platforms, has emerged as a promising approach for procedural skill retention. A 2019 JMIR scoping review found that microlearning interventions demonstrated positive effects on healthcare professionals‘ knowledge, confidence and procedural skills (10), while a 2022 randomized controlled trial confirmed significant improvements in nursing students' learning outcomes and self-efficacy compared to traditional lecture-based methods (11). The theoretical foundation rests on spaced repetition and retrieval practice: skills are rehearsed in short bursts proximal to clinical application, optimizing retention through frequent, low-stakes practice rather than massed training followed by extended disuse.

Despite these methodological advances, few studies have evaluated multi-component, setting-specific intervention bundles that combine leadership accountability, dedicated competency infrastructure and technology-enabled reinforcement. Critically, even fewer have assessed intervention durability under conditions of external stress (e.g., pandemic-driven volume fluctuations, workforce turnover), leaving fundamental questions unanswered about the organizational prerequisites for sustained safety culture transformation.

This study has three distinct objectives: (1) To characterize the dynamic epidemiological and direct economic burden of NSIs from 2018 to 2024 across the three COVID-19 pandemic phases (P1, P2, P3) by quantifying temporal trends, setting-specific incidence rates, and attributable direct medical costs. (2) To evaluate the effectiveness of a multi-component safety intervention bundle implemented in the Infusion Center during the post-COVID-19 pandemic clinical resurgence, assessing whether the bundled strategies (leadership goal-setting, dedicated preceptorship, video-based microlearning) were associated with a sustained risk reduction independent of hospital-wide rebound pressures. (3) To develop and illustrate, as a proof-of-concept, a dynamic early warning system providing real-time, volume-adjusted risk monitoring for hospital management to enable proactive detection of departmental anomalies and evidence-based resource deployment.

## 2 Materials and methods

### 2.1 Study design and setting

A retrospective longitudinal study was conducted from January 2018 to December 2024 at Shanghai Fifth People's Hospital, a large tertiary hospital. The studies involving humans were approved by the Medical Ethics Committee of Shanghai Fifth People's Hospital (Approval No. 075, 2023) and conducted in accordance with the local legislation and institutional requirements, with a waiver of written informed consent to participate from the participants or the participants' legal guardians/next of kin given the retrospective, non-interventional nature of the data. As a non-randomized evaluation of a behavioral public-health intervention, reporting follows the TREND Statement (12).

The study period was categorized into three phases to evaluate the impact of safety interventions against the backdrop of the pandemic: (1) pre-COVID-19 pandemic phase (P1: 2018–2019). (2) intra-COVID-19 pandemic phase (P2: 2020–2022). (3) post-COVID-19 pandemic recovery phase (P3: 2023–2024). The Infusion Center served as the intervention group, while the other 38 clinical departments served as the control group.

### 2.2 Data collection and definitions

Data on all reported NSIs were retrieved from the Department of Hospital Infection Management of Shanghai Fifth People's Hospital. For each reported case, we collected the department, date of injury and the type of sharp instrument involved. To calculate exposure, we obtained the annual number of clinical staff (nursing, medical and logistics personnel) per department. Hospital-wide clinical volume, defined as the sum of annual inpatient visits, was collected as a time-varying covariate for clinical workload.

Staffing headcount (person-years) indexes the number of HCWs at risk per unit time but does not index the number of percutaneous procedures performed. Department-level procedure counts were not recorded centrally in the hospital information system across 2018–2024 and cannot be reconstructed retrospectively for control departments. The per-HCW incidence rate in any department can be decomposed as (procedures per HCW) × (hazard per procedure). Without procedure-level data, these two components cannot be separated, and the per-HCW rate therefore represents a combined occupational burden rather than an intrinsic per-procedure hazard. Under this denominator, the design supports two classes of contrasts, which differ in their susceptibility to the denominator limitation. (1) Cross-sectional between-group contrasts within a period (infusion center vs. controls) represent the total per-HCW occupational NSIs burden of working in the Infusion Center relative to a control department. They are dominated by between-department procedural-volume differences and should not be interpreted as per-procedure hazard ratios. (2) Longitudinal within-group contrasts (infusion center P1 vs. P2 vs. P3; controls P1 vs. P2 vs. P3) hold department identity fixed and thereby eliminate between-department procedural-volume differences.

Procedure-level workload data were available for the Infusion Center alone, obtained internally from the department's performance metric records for 2018–2024. These data supported a within-Infusion-Center per-procedure-unit sensitivity analysis. Year-specific departmental staffing-headcount records were not separately maintained in the hospital information system across the 2018–2024 window. Each department's current staffing headcount was therefore used as a time-invariant offset in the Poisson GLMM, on the basis of institutional confirmation that departmental staffing levels were stable across the study period.

**Figure 1:**
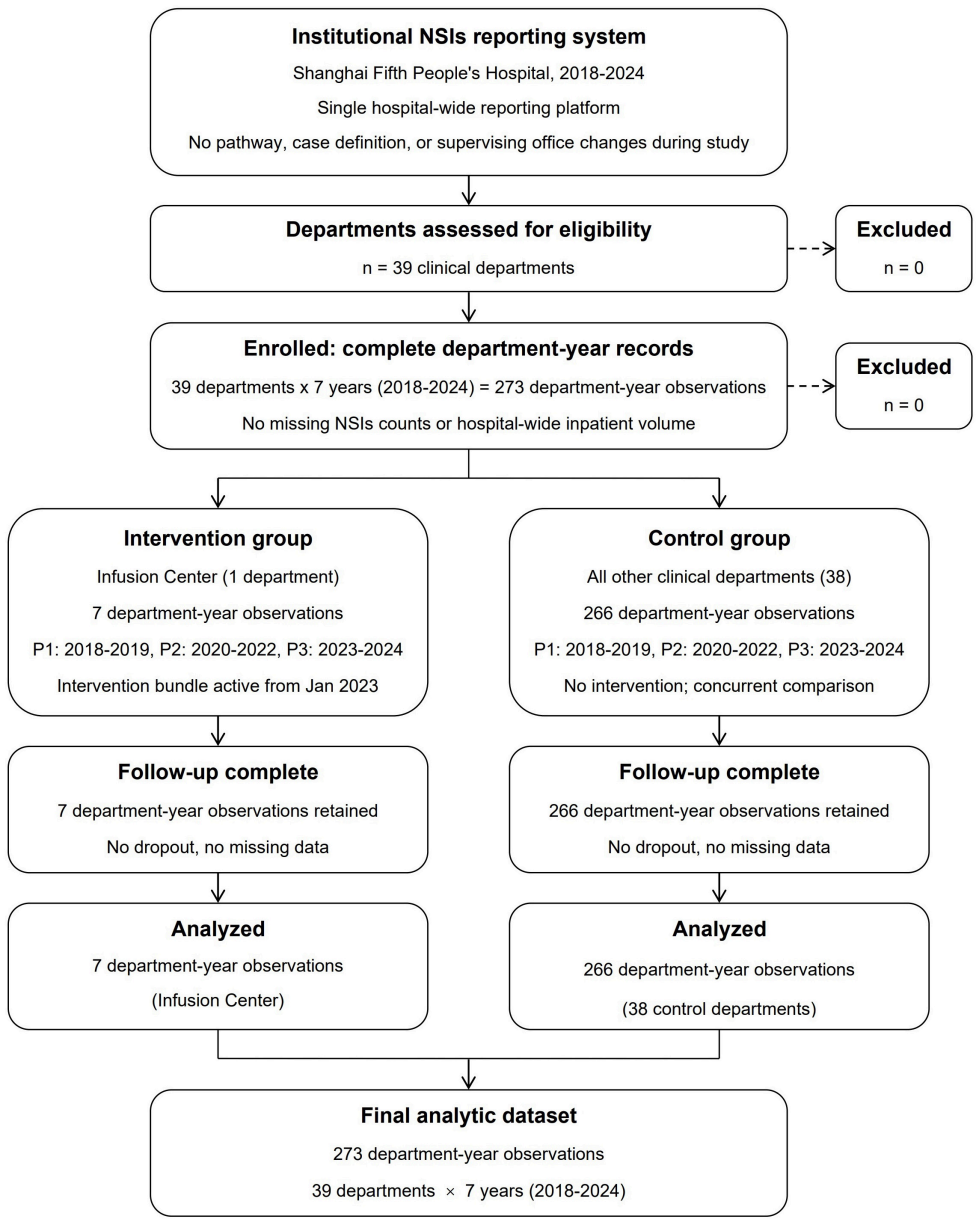
Inclusion and exclusion flow of the study cohort.

All reports were captured through a single hospital-wide NSIs reporting platform managed by the Department of Hospital Infection Management throughout 2018–2024. No changes in reporting pathway, case definition, or supervising office occurred during the study window. All 39 clinical departments contributed continuous NSIs incident records for every calendar year from 2018 through 2024, yielding 39 × 7 = 273 department-year observations with no exclusions. No missing values were observed in the key variables (department, year, NSIs count, hospital-wide inpatient volume) and no imputation was required (Figure 1).

The safety intervention bundle was implemented in a phased approach synchronized with P3. Administrative leadership was formalized in January 2023 through the establishment of “zero-NSI” performance metrics added to the head-nurse role, with monthly rate reporting to hospital leadership and annual appraisal incorporation. The component operated at the organizational level through leadership accountability rather than direct front-line training, and was active throughout P3. The dedicated preceptorship was also introduced in January 2023, pairing newly hired and rotating nurses with trained senior-nurse preceptors who delivered bedside coaching sessions during live clinical shifts using a standardized competency checklist, with a targeted 2-5 sessions per nurse per month. It expanded from pilot scheme to full departmental coverage through H1 2023, with full operation from Q3 2023 onwards. Finally, the digitalized microlearning curriculum comprising eight short video modules on error-prone needle-handling scenarios was delivered via a self-paced cloud platform with individual nurse logins, with completion mandatory for newly hired and rotating nurses within the first week in the department. It was launched in November 2024 and active for approximately 2 months of P3 to standardize long-term compliance (intervention details presented in Supplementary Table S1) (13).

### 2.3 Assessment of disease and economic burden

The economic burden of NSIs was quantified using direct medical costs, specifically the sum of laboratory testing and PEP medication expenses. These data were extracted directly from the hospital information system for all reported cases between 2018 and 2024. The average direct cost per NSI case was calculated as the mean cost across the entire hospital cases to ensure a stable and representative unit cost for subsequent analysis. This constitutes a partial direct-cost analysis and does not capture indirect costs (time off work, productivity loss), counselling or psychological follow-up, costs of long-term hepatitis B virus (HBV)/hepatitis C virus (HCV)/treponema pallidum (TP)/human immunodeficiency virus (HIV) seroconversion and lifetime treatment, legal/administrative costs, or the cost of the intervention itself (preceptor time, training platform, leadership oversight time); all estimated avoidance should therefore be interpreted as lower bounds.

To evaluate the financial impact of the safety intervention bundle in the Infusion Center, a counterfactual cost-avoidance analysis was employed. This approach estimated the “avoided cost” by comparing the actual observed NSIs cases in P3 against an expected number of NSIs cases. The expected cases were calculated by applying the P1 incidence rate to the P3 exposure volume, assuming that in the absence of the interventions, the Infusion Center would have reverted to its historical high-risk status. This counterfactual was chosen as it provided a conservative estimate of the interventions' economic value by using the department's own historical performance as the reference point.

### 2.4 Statistical analysis

The longitudinal data were analyzed using a generalized linear mixed model (GLMM) with a Poisson distribution. The model utilized a random intercept for “department” to adjust for repeated measures over the 7-year period. To formally assess whether additional overdispersion beyond that captured by the random effects warranted a negative binomial specification, both Poisson and negative binomial GLMMs were estimated using identical fixed-effects structures. The negative binomial overdispersion parameter was non-significant (lnα = −13.89, SE = 507.15, *p* = 0.978) and both models converged to identical log-likelihoods (−336.66). Formal model selection criteria confirmed the Poisson specification as optimal: AIC (689.31 vs. 691.31) and BIC (718.19 vs. 723.80) were both lower for the Poisson GLMM. The significant random intercept variance (σ^2^ = 0.351, 95% CI: 0.169–0.729, LR test vs. fixed-effects Poisson: χ^2^ (1) = 37.07, *p* < 0.001) confirmed that between-department heterogeneity was adequately captured by the random effects structure without requiring an additional dispersion parameter. Pearson residuals were examined against model-predicted values and against calendar year, no systematic patterns or temporal heteroscedasticity were detected. The Poisson GLMM with random intercepts was therefore adopted as the final model.

Fixed effects included setting (Infusion Center vs. control clinical departments), study period (P1, P2 and P3) and their interaction term. The model adjusted for hospital-wide annual clinical volume (total inpatient admissions) as a time-varying covariate to control for the impact of hospital-wide clinical service recovery and utilized log-transformed clinical staff count as an offset variable to model the incidence rate and estimate incidence rate ratios (IRRs). To deconstruct the interaction, we performed *post-hoc* simple effect analyses: (1) longitudinal contrasts (within-group change over time) and (2) cross-sectional contrasts (between-group differences per period). Under this specification, the setting × period interaction term is, by construction, a difference-in-differences (DiD) estimator: it quantifies the change in log-incidence in the Infusion Center from one period to another, net of the hospital-wide secular trend captured by the control group. The interaction IRR should therefore be interpreted as an association attributable to exposure to the Infusion Center's setting during the relevant period after removing common secular effects, rather than as an individually randomized causal contrast.

A within-Infusion-Center sensitivity analysis using procedural workload as an alternative to staffing as the exposure offset was conducted. Annual procedural workload scores for 2018–2024 were obtained from the Infusion Center's performance metric records. The metric was a weighted composite of seven activity types (medication intake 0.2, medication preparation 0.3, intravenous infusion 1.5, fluid replacement 0.2, needle removal 0.3, ambulation rounds 0.1, supplementary injection 1.5, push injection 0.4) and summed annually. We fitted a single-level Poisson regression on the seven Infusion Center annual observations with NSIs count as outcome, period (P1, P2, P3) as the fixed effect, and log (workload) as the offset, generating per-procedure-unit IRRs. Comparable workload data were not available for control departments, the sensitivity analysis was therefore restricted to the Infusion Center and the main multilevel GLMM with setting × period interaction retained the per-HCW (staffing) denominator as its primary specification.

All statistical analyses were performed using Stata/MP version 14.1 (StataCorp, College Station, TX, USA). Statistical significance was defined as *p* < 0.05 (two-tailed).

### 2.5 Construction of the dynamic early warning system

A proof-of-concept dynamic early warning system was developed based on statistical process control (SPC) principles. Using the GLMM parameters, we generated department-specific UCLs. UCLs were computed as conditional prediction intervals based on each department's estimated random intercept (best linear unbiased predictor), incorporating both fixed-effect (period × volume) and random-effect (department-specific baseline risk) components. These limits were dynamic, as they adjusted for each department's historical risk (random effects) and real-time fluctuations in hospital-wide volume (fixed effects).

We established two alarm thresholds:

- Level-I Warning (Yellow): observed NSIs exceeding the 95% prediction interval.
- Level-II Action Alarm (Red): observed NSIs exceeding the 99% prediction interval. The system's utility was validated by analyzing alarm triggers during P3.

### 2.6 Contextual key-informant interviews

To complement the quantitative analysis, planned key-informant interviews were conducted with the Infusion Center head nurse during routine site visits in 2024. The interviews had two pre-specified objectives: (1) to elicit detailed information on the implementation of the three safety-intervention components and the team's operational pursuit of the “zero-NSI” target, and (2) to retrieve longitudinal procedural workload data for the Infusion Center for the 2018–2024 study window, which were subsequently used as the per-procedure denominator in the within-Infusion-Center sensitivity analysis. The interview content was recorded as written field notes by the study team and summarized as descriptive contextual observations. We did not perform formal qualitative coding, audio-recorded transcription, or independent inter-rater verification. The contextual observations were therefore presented as informant-derived descriptive findings rather than as the output of a formal qualitative study.

## 3 Results

### 3.1 Baseline characteristics

Throughout the results, we clearly distinguish count (absolute NSIs cases), incidence rate (cases per 100 person-years), predicted value (model-based marginal mean at specified covariate values), and incidence rate ratio (IRR) (multiplicative incidence rate ratio between groups or periods).

Table 1 summarizes the absolute frequencies, exposure person-years and crude incidence rates of NSIs stratified by clinical setting and study period. In P1, the crude incidence rate in the Infusion Center was nearly 20 times higher than that of the control group (72.5 (95% CI: 48.55–104.12) vs. 3.67 (95% CI: 2.92–4.55) per 100 person-years, respectively). This underscored the Infusion Center's historical status as a high-risk environment for percutaneous exposures. Longitudinal observation revealed diverging trends between the two groups. In the control group, the crude NSIs rate decreased during P2 to 2.51 (95% CI: 2.0–3.1) per 100 person-years, before rebounding to 3.58 (95% CI: 2.84–4.45) per 100 person-years in P3. In stark contrast, the Infusion Center presented a continuous and steep decline in crude NSIs rates across the study periods. The rate fell by more than half during P2 (23.33 (95% CI: 12.76–39.15) per 100 person-years) and continued to drop in P3 to 22.5 (95% CI: 10.29–42.71) per 100 person-years.

**Table 1:** Baseline characteristics and descriptive incidence rates.

Study period	Clinical setting	Total NSIs (*n*)	Exposure (person-years)	Crude incidence rate (per 100 person-years) (95% CI)	Laboratory testing expense (RMB)	PEP medication expense (RMB)	Total expense (RMB)
P1	Other 38 clinical departments	83	2,262	3.67 (2.92–4.55)	54,220	1,890	56,110
P2	Other 38 clinical departments	85	3,393	2.51 (2.0–3.1)	48,380	3,780	52,160
P3	Other 38 clinical departments	81	2,262	3.58 (2.84–4.45)	53,705	1,860	55,565
P1	Infusion Center	29	40	72.5 (48.55–104.12)	17,450	0	17,450
P2	Infusion Center	14	60	23.33 (12.76–39.15)	7,223	300	7,523
P3	Infusion Center	9	40	22.5 (10.29–42.71)	3,481	0	3,481
P1	Hospital-wide	112	2,302	4.87 (4.01–5.85)	71,670	1,890	73,560
P2	Hospital-wide	99	3,453	2.87 (2.33–3.49)	55,603	4,080	59,683
P3	Hospital-wide	90	2,302	3.91 (3.14–4.81)	57,186	1,860	59,046

### 3.2 Mixed-effects regression analysis of NSIs risk factors

To account for the longitudinal clustering of data, a mixed-effects Poisson regression model (Poisson GLMM) was employed, confirmed as the optimal distributional specification over a negative binomial alternative based on model comparison. Despite its lack of statistical significance in the current sample, total hospital clinical volume was retained as a structural covariate *a priori* to ensure the dynamic early warning system effectively adjusted for fluctuations in healthcare system workload, thereby preventing clinical surges from being misinterpreted as systemic protocol failures.

#### 3.2.1 Baseline and environmental factors

The model revealed that the Infusion Center was historically a high-risk setting, with a baseline NSIs risk 22.43 times higher than that of the control group (IRR = 22.43, 95% CI: 6.40–78.61, *P* < 0.001) during P1. Interestingly, while hospital-wide clinical volume showed a positive trend with NSIs occurrences, it did not reach statistical significance as an independent predictor in the multivariable environment (IRR = 0.47, *p* = 0.386), suggesting that setting-specific factors and pandemic-related phase shifts were the primary drivers of risk fluctuations.

#### 3.2.2 Longitudinal trends in control departments

In the other 38 clinical departments, NSIs risk followed a distinct “rebound” pattern. Compared to P1, the risk of NSIs significantly decreased by 43.3% during P2 (IRR = 0.57, 95% CI: 0.34–0.96, *p* = 0.033). However, the risk in these departments rebounded to P1 levels in P3, showing no significant difference from P1 (IRR = 1.06, 95% CI: 0.74–1.51, *p* = 0.753).

#### 3.2.3 The interaction effect: the Infusion Center's divergent path

The core finding of this study was the significant interaction between clinical setting and study period. During P2, the Infusion Center showed a significantly greater reduction in risk compared to the control group's trend (interaction IRR = 0.47, 95% CI: 0.23–0.95, *p* = 0.037).

Most importantly, this protective trend intensified during P3. The interaction term for the Infusion Center during P3 was 0.32 (95% CI: 0.14–0.71, *p* = 0.005), indicating that the Infusion Center's NSIs risk was 68.2% lower than what would have been expected if it had followed the hospital-wide rebound trend.

### 3.3 Simple effect analysis of the divergent risk trajectories

To deconstruct the significant interaction between clinical setting and study period, a *post-hoc* simple effect analysis was conducted to evaluate both between-group and within-group risk disparities (Table 2).

**Table 2:** IRRs of NSIs across clinical settings and study periods (simple effect analysis).

Comparison	IRR	Std. Err.	95% CI	*p*
Panel A: cross-sectional analysis (between-group differences), reference: control group				
P1: infusion Center vs. control group	22.43	14.35	6.40– 78.61	< 0.001
P2: infusion Center vs. control group	10.57	7.06	2.86–39.15	0.0004
P3: infusion Center vs. control group	7.13	4.97	1.82–27.98	0.005
Panel B: longitudinal analysis (within-group changes over time), reference: P1 baseline				
Control group				
P2 vs. P1	0.57	0.15	0.34–0.96	0.033
P3 vs. P1	1.06	0.19	0.74–1.51	0.753
Infusion center				
P2 vs. P1	0.27	0.1	0.12–0.58	0.0007
P3 vs. P1	0.34	0.13	0.16–0.73	0.0006

Cross-sectional comparisons (Panel A) revealed that while the adjusted risk of NSIs in the Infusion Center remained consistently higher than that in the control group across all study periods, reflecting the inherently high-frequency nature of percutaneous exposures in this setting, the relative risk disparity narrowed substantially over time. In P1, the Infusion Center exhibited a 22.43-fold higher risk of NSIs compared to the control group (*p* < 0.001). During P2, this relative risk decreased to 10.57 (*p* = 0.0004). Notably, during P3, the risk ratio further contracted to 7.13 (*p* = 0.005). This progressive reduction indicated a sustained protective trend within the Infusion Center that mitigated the expected surge in exposures relative to the other 38 clinical departments.

Longitudinal within-group analyses (Panel B) further elucidated these strikingly divergent trajectories. Within the control group, the adjusted NSIs incidence rate significantly decreased during P2 compared to P1 (IRR = 0.57, 95% CI: 0.34–0.96, *p* = 0.033). However, during P3, this rate rebounded entirely, showing no statistical difference from P1 (IRR = 1.06, 95% CI: 0.74–1.51, *p* = 0.753). Conversely, the Infusion Center presented a sharp and sustained decline in absolute NSIs risk. Compared to its own P1, the incidence rate dropped significantly during P2 (IRR = 0.27, *p* = 0.0007) and crucially, did not rebound during P3. Instead, the Infusion Center's risk remained significantly reduced at approximately one-third of its P1 level (IRR = 0.34, 95% CI: 0.16–0.73, *p* = 0.0006). This suggested that the safety improvements in the Infusion Center were sustained independently of the hospital-wide clinical resurgence.

The within-Infusion-Center sensitivity analysis confirmed and strengthened the longitudinal findings on a procedural-volume-adjusted denominator. Annual workload scores for the Infusion Center totalled 173,706 in P1, 175,063 in P2 and 427,441 in P3 (period means), so the P3/P1 workload ratio was 2.46, indicating that per-HCW procedural volume was approximately 2.5-fold higher in P3 than P1. With log (workload) as the offset, the per-procedure-unit Poisson model returned IRR P2 vs. P1 = 0.32 (95% CI: 0.17–0.60, *p* < 0.001), IRR P3 vs. P1 = 0.13 (95% CI: 0.06–0.27, *p* < 0.001) and IRR P3 vs. P2 = 0.40 (95% CI: 0.17–0.91, *p* = 0.03). On the same seven annual observations the per-HCW Poisson model (offset = log staffing) returned IRR P2 vs. P1 = 0.32 (95% CI: 0.17–0.61) and IRR P3 vs. P1 = 0.31 (95% CI: 0.15–0.66). The per-procedure-unit P3 vs. P1 IRR was therefore approximately one third of the per-HCW IRR, indicating a 87% reduction in NSIs hazard per workload-score unit relative to a 69% reduction per HCW. Because P3 procedural workload was 2.5-fold higher than P1 while NSIs counts fell, the per-HCW analysis used in the main GLMM was conservative for the headline post-pandemic contrast. The P2 vs. P1 IRRs were essentially identical under the two denominators (0.319 vs. 0.322), reflecting the near-identical P1 and P2 workload (P2/P1 = 1.01). The divergence between the two analyses was concentrated in the P3 contrast, exactly as the workload data predict.

### 3.4 Visualizing the divergent trajectories of NSIs risk (marginal effects analysis)

**Figure 2:**
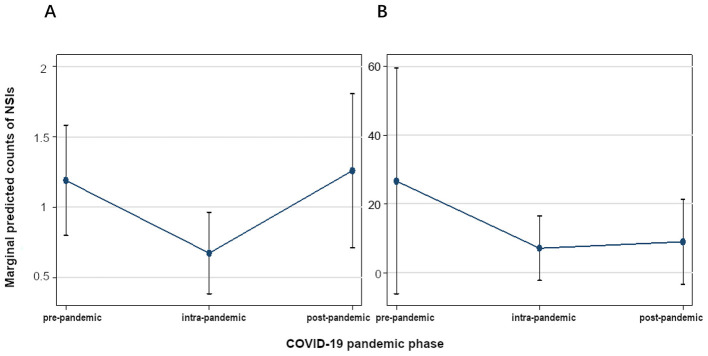
Marginal predicted counts of NSIs across COVID-19 pandemic phases. **(A)** The control group. **(B)** The Infusion Center. The facet grid illustrates the absolute risk evolution for the control group and the Infusion Center. Predictions are derived from the Poisson GLMM, adjusting for temporal hospital volume and standardizing the staff size (offset) at the mean level. Independent Y-axis scales are applied to clearly display the non-parallel trends (V-shaped rebound vs. L-shaped sustained reduction) despite the vast difference in baseline counts. A nominal CI lower bound below zero for small subgroups with wide intervals is an artefact of the Wald approximation and should be interpreted as effectively zero. Adjusted predictions of is_infusion#period with 95% CIs.

To further illustrate the interaction between clinical setting and study period, we calculated the adjusted marginal predicted means of NSIs occurrences while holding other covariates at their means. The resulting marginal effects plot (Figure 2) clearly revealed the divergent trajectories of NSIs risks between the two groups.

In the control group, the adjusted predicted mean of NSIs followed a distinct U-shaped rebound pattern. The predicted mean dropped from 1.19 (95% CI: 0.80–1.58) during P1 to 0.67 (95% CI: 0.39–0.96) in P2, before rebounding to 1.26 (95% CI: 0.71–1.81) in P3. This rebound in P3 slightly exceeded the P1 level, reflecting the hospital-wide return to normal clinical activity.

In sharp contrast, the Infusion Center presented a pattern of sustained risk reduction. The adjusted predicted mean was highest in P1 at 26.69 (95% CI: −6.13–59.52). Following the implementation of safety interventions, the predicted mean plummeted to 7.13 (95% CI: −2.25–16.52) in P2 and remained reduced at 8.99 (95% CI: −3.35–21.33) during P3. Confidence intervals (CI) were Wald-type (Gaussian) approximations on the predicted count scale, for small subgroups with wide intervals, the lower bound may nominally fall below zero, which was not possible for count data. The true count was bounded at zero and point estimates were unaffected.

Crucially, while the control group's predicted NSIs risk in P3 surpassed its P1 baseline, the Infusion Center's P3 risk remained at approximately one-third of its P1 level. The visual divergence in P3 suggested that the safety interventions in the Infusion Center attenuated the systemic pressure of increased patient volume, avoiding a return to the high-risk baseline observed in P1.

### 3.5 Application of the dynamic early warning system

To translate the statistical findings into actionable infection control management, the dynamic early warning system was applied to P3 to identify institutional anomalies driven by patient volume recovery. Table 3 summarizes the alarms triggered across all 39 clinical departments during this 2-year phase (comprising 78 department-years).

**Table 3:** Summary of early warning alarms triggered during P3.

Clinical setting/department	Total observation years in P3	Level-I warnings (yellow)	Level-II action alarms (red)
Infusion Center	2	0	0
**Control group (high-risk outliers)**			
Endocrinology	2	0	2
Respiratory medicine	2	0	2
Thoracic surgery	2	0	2
Operating room	2	1	1
**Control group (other clinical departments)**			
All other 34 clinical departments	68	2	15
Hospital-wide	78	3	22

Within the control group, the system detected widespread breaches of the personalized UCLs. Specifically, non-intervention departments generated 3 Level-I warnings (exceeding the 95% UCL) and 22 Level-II action alarms (exceeding the 99% UCL). Notably, several high-stress departments, including endocrinology, respiratory medicine and thoracic surgery, triggered recurrent Level-II alarms in both years of P3. This indicated a systemic breakdown in safety protocols amidst the hospital-wide clinical surge.

In stark contrast, the Infusion Center triggered zero alarms during the entire P3, maintaining a completely “normal“ status. The observed NSIs in the Infusion Center remained strictly below its volume-adjusted UCL. This surveillance outcome was consistent with sustained effectiveness of the localized safety interventions, illustrating that observed counts remained within the volume-adjusted envelope even under the pressure of systemic volume recovery. External validation of the thresholds in independent hospital data is required before any operational alarm threshold can be recommended.

**Figure 3:**
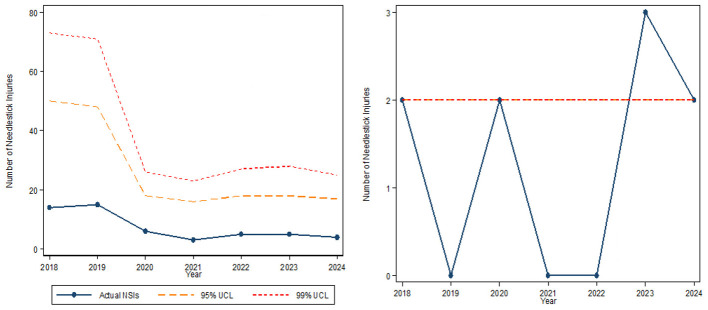
Dynamic surveillance of NSIs counts against department-specific 95% and 99% UCLs during P3. **(A)** The Infusion Center. **(B)** The department of endocrinology. The dashed lines represent the dynamic UCLs. The 95% UCL indicates the level-I warning limit and the 99% UCL indicates the level-II action alarm limit. Independent y-axis scales are applied to Panels A and B because the Infusion Center's absolute counts and dynamic UCLs are an order of magnitude higher than those of the endocrinology department; a shared axis would compress Panel B to visual flatness and obscure the P3 UCL breach that is central to the surveillance demonstration.

The temporal dynamics of these surveillance outcomes and the sensitivity of the dynamic thresholds are further visualized in Figure 3. Panel A illustrates the longitudinal trajectory of the Infusion Center. Despite a hospital-wide resurgence in clinical volume during P3, the observed incidence of NSIs consistently tracked below both the 95% UCL and the 99% UCL, visually confirming the sustained effectiveness of the localized safety interventions.

In contrast, Panel B provides a representative control chart for a high-risk outlier department (endocrinology). The actual NSIs occurrences in this setting can be seen sharply breaching the 99% UCL during P3. Because these control limits natively adjusted for baseline departmental risk and fluctuations in hospital-wide volume, this visual breach confirmed a true systemic protocol failure rather than a statistical artifact of increased patient load.

### 3.6 Economic burden and cost avoidance analysis

To quantify the economic benefit of the targeted interventions in the Infusion Center, a counterfactual cost-avoidance analysis was conducted. Over the study periods, the average direct medical cost per NSI case, encompassing laboratory testing for bloodborne pathogens and PEP medication, was calculated at 638.83 RMB.

Assuming the Infusion Center had maintained its P1 incidence rate (72.5 per 100 person-years) without localized safety interventions, the expected number of percutaneous exposures during P3 would have reached 29 cases. However, the actual observed occurrences were limited to only nine cases. This indicated that the comprehensive safety interventions were associated with 20 fewer NSIs cases within this single high-risk department during P3 than projected under the counterfactual. Applying the hospital-wide mean per-case direct cost, it corresponded to a direct medical cost avoidance of 12,776.68 RMB, which represented a lower-bound estimate of direct medical costs averted, excluding indirect productivity loss, psychological burden, long-term seroconversion treatment costs, administrative costs, and the cost of the intervention itself. It did not constitute a cost-effectiveness or cost-benefit analysis.

### 3.7 Contextual observations from key-informant interviews

Three contextual observations were derived from the planned key-informant interviews with the Infusion Center head nurse and presented here as descriptive findings.

First, leadership-driven goal specificity. The Infusion Center's new head nurse explicitly adopted a “zero-NSI” target as a core performance metric for the department, with monthly NSIs-rate reporting incorporated into routine departmental management and annual appraisal documentation. The informant reported that the control departments lacked comparably explicit NSIs-specific goals, with department directors citing general infection-control awareness rather than prioritizing NSIs reduction as a distinct, measurable objective.

Second, dedicated competency infrastructure. The Infusion Center deployed designated senior-nurse preceptors who conducted live bedside demonstrations of correct sharps-handling and proper sharp disposal technique during routine clinical shifts, allowing newly hired and rotating nurses to observe and practice under direct supervision. The informant reported that the control departments relied on traditional buddy-system training and generic hospital orientation lectures covering sharp-safety rules without procedural integration.

Third, video-based microlearning. A cloud-based curriculum of eight short video modules depicting error-prone needle-handling scenarios (e.g., “needle bobbing while infusion dressing adhered to glove”, “reaching into sharps container while handling needle”) was deployed in November 2024 to support just-in-time reinforcement during shift breaks. The informant reported that the control departments had no comparable just-in-time reinforcement mechanism. We stated for transparency that this component was active for only 2 months within the P3 observation window and was therefore unlikely to have contributed materially to the P3 quantitative findings. It was included here as a descriptive observation about the bundle's design rather than as an attribution of effect.

## 4 Discussion

This seven-year longitudinal study is consistent with the hypothesis that systematic, setting-specific safety interventions can achieve sustained reductions in NSIs risk even amid healthcare system volatility. The Infusion Center achieved 73% risk reduction during P2 and retained 66% reduction throughout P3, while the other 38 clinical departments rebounded to P1 levels (IRR = 0.34 vs. 1.06, respectively). Two interpretive observations follow from these results. First, because the model offset is staffing headcount (person-years) rather than the absolute number of percutaneous procedures, the 22.43-fold P1 IRR should be interpreted as the cumulative per-HCW environmental risk of working in the Infusion Center relative to the control departments rather than as an elevated per-procedure hazard, since infusion nurses inherently perform substantially more needle-related procedures per shift than general-ward staff. Second, the significant interaction term in P3 (IRR = 0.32, 95% CI: 0.14–0.71, *p* = 0.005) is consistent with the safety-intervention bundle attenuating the NSIs risk increase that would otherwise have accompanied the post-pandemic clinical-volume rebound, although the observational, single-center design precludes a strict causal claim.

The dynamic surveillance system identified 25 alarm cases in the control departments while the intervention site maintained zero alarms, a pattern consistent with sustained protective effects that were not explained by hospital-wide volume fluctuations.

Our baseline finding of 22.43-fold higher NSIs risk in the Infusion Center aligns with the evidence identifying outpatient infusion facilities as high-risk environments due to high patient throughput, time pressure and procedure-intensive workflows (5). A 7-year retrospective study in an oncology setting found that blood collection (26.5%) and waste collection (18.3%) were the primary causes of NSIs, with nurses accounting for 62% of all exposures (14).

The 43% NSIs reduction in the control departments during P2, followed by complete rebound, mirrors an international surveillance pattern. Diktas et al. (9) reported that NSIs rates in Turkish hospitals declined from 27.65% (2019) to 21.4% (2020) during pandemic lockdowns. However, most pandemic- era studies lacked extended follow-up. Our 7-year window reveals that pandemic-driven behavioral changes are transient without institutionalized safety practices, while the Infusion Center's sustained reduction suggests that proactive, setting-specific interventions can achieve durable results.

While the Infusion Center showed substantially lower NSIs rate during P2, this coincided with pandemic-related behavioral adaptations observed hospital-wide (control P2 reduction: IRR = 0.57). The primary contribution of the P3 safety-intervention bundle is therefore the prevention of post-pandemic rebound, evidenced by the significant interaction term (IRR = 0.32, *p* = 0.005) against a backdrop of complete rebound in the control departments. The three contextual observations from the key-informant interviews are interpretable in the light of established safety-science literature. The leadership-driven “zero-NSI” target observed in the Infusion Center is consistent with evidence that concrete, measurable goals predict injury outcomes more strongly than generic safety attitudes (15, 16). The absence of comparable goal specificity in the control departments offers a plausible explanation for their post-pandemic rebound once external pandemic-driven threat perception receded. The dedicated competency infrastructure observed in the Infusion Center is consistent with evidence that simulation-based medical education with deliberate practice is superior to traditional apprenticeship-based clinical education in skill acquisition and retention (17). The control departments' reliance on traditional buddy-system training and generic orientation lectures represents a decontextualized approach prone to poor knowledge transfer under multitasking pressure (18). The video-based microlearning curriculum observed in the Infusion Center is concordant with evidence that short, modular digital learning supports knowledge retention and procedural-skill confidence in HCWs (10, 11). However, given its November 2024 deployment, this component is unlikely to have contributed materially to the P3 quantitative finding and is interpreted as a forward-looking element of the bundle's design rather than as an attribution of effect.

These findings align with implementation science emphasizing that durable behavior change requires embedding safety into operational muscle memory through contextual practice, real-time feedback and leadership accountability (11, 19), not merely knowledge dissemination.

Our findings challenge assumptions that procedurally intensive settings must tolerate elevated injury rates. The Infusion Center's achievement suggests that baseline risk does not dictate intervention ceiling. Rather than diffuse hospital-wide campaigns, concentrated investment in empirically identified hotspots may yield superior returns. The dynamic surveillance system provides a scalable tool for such prioritization by flagging departments exhibiting risk disproportionate to workload. The 25 alarms triggered in control departments during P3 represent actionable targets for root-cause analysis and focused remediation.

Traditional NSIs prevention has centered on cognitive interventions including didactic education emphasizing sharp safety rules, poster campaigns and annual competency checklists. Our results suggest these are necessary but insufficient. Sustained behavior change requires embedding safety into operational practice through contextual skill development, real-time feedback and leadership-enforced accountability (19, 20). Healthcare infection control should adopt analogous strategies: designating safety champions in high-risk settings, incorporating sharp technique evaluation into preceptorship milestones and institutionalizing near-miss debriefs as standard practice.

This study's 7-year observation window enables causal inference approximation through difference-in-differences analysis controlling for secular trends and setting-specific confounders. The volume-adjusted surveillance system addresses a persistent challenge in healthcare safety, distinguishing signal from noise when incident counts fluctuate with clinical volume.

Behavioral bundle of the kind evaluated here is complement to, not substitute for, engineering controls. International evidence consistently identifies safety-engineered devices (SEDs) as among the most effective NSIs-reduction interventions, with a meta-analysis by Tarigan and colleagues reporting substantial pooled risk reductions following SEDs introduction (21). No new SEDs were introduced in the Infusion Center during the study period. The bundle studied here is a behavioral overlay on an engineering-controls baseline mandated by Chinese national occupational-safety regulations at the hospital. Future studies should evaluate whether combined SEDs-plus-behavioral intervention bundle outperforms either component administered alone.

The economic burden captured in the present analysis is direct medical cost only, comprising laboratory testing for bloodborne-pathogen serology and PEP medication recorded in the hospital information system for each reported case. The 12,776.68 RMB figure for the Infusion Center P3 should therefore be interpreted as a lower-bound estimate of avoided direct medical cost under the counterfactual and it does not constitute a cost-effectiveness or cost-benefit analysis. The per-case direct medical cost observed in this study is broadly consistent with the international range reported by Cooke and Stephens (4). Substantial categories of indirect cost are not captured in this figure and should be considered when interpreting the broader burden of NSIs: (1) productivity loss during exposure assessment, follow-up visits and possible work restriction; (2) psychological burden during the seroconversion-monitoring window (typically 3–6 months for HBV/HCV/TP/HIV per occupational-health protocols), which carries documented anxiety, sleep disturbance and reduced job satisfaction; (3) long-term costs of chronic infection in the small fraction of HCWs who would seroconvert (HBV/HCV/TP/HIV lifetime treatment, in the order of tens to hundreds of thousands of RMB per case in the Chinese setting); (4) administrative and legal/insurance costs associated with documenting the exposure event; and (5) the cost of the intervention bundle itself (preceptor staff time, training-platform fees and leadership oversight time). The international literature consistently estimates that direct costs typically represent a minority of the total economic burden of NSIs, with productivity loss, psychological burden and follow-up dominating the total Figure (4). The true avoided burden in this study is therefore likely several-fold higher than the reported direct figure.

One specific feature of the Chinese institutional context further shapes the per-case direct-cost figure reported here. At the study hospital, PEP is administered on a case-by-case basis: for each reported NSI, the hepatitis B serological status of both the injured HCW and the source patient is assessed at the time of injury, and HBV PEP comprising hepatitis B vaccine and/or hepatitis B immune globulin (HBIG) is then administered in line with CDC PEP guidelines. The same case-by-case serology-driven approach applies for HCV, TP, and HIV. The hepatitis B vaccine, when administered as PEP to HCWs, is included in China's National Immunization Program (NIP) and provided free of charge and it therefore does not enter the hospital information system as a billable direct medical cost. In contrast, HBIG is not included in the NIP and is billed through the standard hospital pharmacy system, as are HCV, TP, and HIV PEP regimens when administered. In the present dataset, hepatitis B vaccine PEP was administered to 8 of the 29 reported P1 Infusion Center cases (typically when source-patient hepatitis B serological status was unclear) and to 0 of the 9 P3 cases, but neither HBIG nor HCV/TP/HIV PEP was administered in any reported case in either period. The reported zero PEP medication cost in Table 1 therefore reflects two coexisting factors: the NIP subsidy of HBV vaccine PEP (which removes administered HBV-vaccine doses from the hospital's billable direct-cost stream) and the absence of clinical indication for the billable PEP components (HBIG, HCV/TP/HIV regimens) for these specific cases. The reported per-case direct cost in P3 (386.8 RMB in Infusion Center, 656.1 RMB hospital-wide) is therefore dominated by laboratory serology testing, and is a lower-bound estimate that excludes the NIP-borne portion of HBV vaccine PEP. In settings without a comparable subsidy program, or in cases where source patient serological status warrants HBIG or HCV/TP/HIV PEP, the per-case direct cost would be substantially higher.

The GLMM-derived UCLs used for the early warning system differ in three principled ways from the standard c-chart and u-chart approach to statistical process control in healthcare (22). First, they are conditional prediction intervals that incorporate a department-specific random intercept, so a department with a historically higher baseline is not systematically flagged as anomalous at every measurement. Second, the fixed-effect structure permits adjustment for hospital-wide workload (inpatient volume), so a surge in clinical activity is not misclassified as a protocol failure. Third, standard c- and u-charts assume independence across time and do not natively accommodate either adjustment. We present the GLMM-based approach as better suited to the clustered, workload-dependent structure of departmental NSIs counts, not as superior in every respect. Traditional SPC remains a valid comparator for single-department, stationary processes.

NSIs are widely under-reported in the Chinese setting (2), and reporting behavior could in principle vary across pandemic phases and in response to reporting system stability and departmental interventions. Three observations are relevant. First, Shanghai Fifth People's Hospital operated a single hospital-wide reporting platform under unchanged custodianship throughout 2018–2024, so system-level reporting pathways cannot explain between-phase differences. Second, the P2 reduction in control departments is consistent both with genuine behavioral change (intensified PPE, deferral of non-essential sharp procedures) and with a plausible increase in under-reporting during pandemic peaks, and we do not claim a directional interpretation. Third, a “zero-NSI” performance metric might in principle suppress reporting in the Infusion Center, however, the data do not support this reporting pattern shift. The Infusion Center's mean direct cost per reported case declined from 601.7 RMB in P1 (17,450 RMB/29 cases) to 386.8 RMB in P3 (3,481 RMB/9 cases), a direction opposite to what selective suppression of minor cases would produce. PEP medication, which is initiated only when a reported case crosses a defined clinical severity threshold, was not required in any reported Infusion Center case in either period (0 of 29 in P1; 0 of 9 in P3), also inconsistent with a shift toward clinically serious cases. As an order-of-magnitude check, the Infusion Center's P3 per-case cost (386.8 RMB) is within the same order as the hospital-wide P3 mean (656.1 RMB), indicating no anomaly in the reporting pipeline specific to the Infusion Center. Interview with the head nurses confirmed that the reporting pattern in the Infusion Center remained consistent before and after the interventions, with supervisory monitoring enforced for every incident. However, two limitations of this argument should be acknowledged: the P3 case count (*n* = 9) makes the per-case cost mean sensitive to individual case draws, for which the PEP-utilization check is a more robust clinical-threshold-based alternative; and the argument rules out selective suppression of minor cases but cannot exclude uniform across-severity under-reporting, which would produce no detectable case-mix signature. Residual reporting bias nevertheless remains as a genuine limitation.

Regression to the mean (RTM) is a ubiquitous phenomenon in repeated observational data and warrants explicit consideration given the Infusion Center's extreme P1 baseline (23). RTM alone, however, would predict partial regression in P2 followed by drift back toward the baseline in P3. The observed pattern is not of this form: the Infusion Center's P3 incidence remained at approximately one-third of its P1 level (IRR P3 vs. P1 = 0.34, *p* = 0.0006), while the control group rebounded essentially to its P1 level (IRR = 1.06, *p* = 0.753). The significant setting × period interaction (IRR = 0.32, *p* = 0.005) therefore captures a group-specific divergence that RTM alone does not parsimoniously reproduce, although it cannot be excluded as a partial contributor to the P1–P2 decline.

A primary limitation of this longitudinal study is the reliance on staffing headcount (person-years) as the exposure denominator. While standard for occupational surveillance, this metric does not account for the drastic differences in procedural intensity between departments. The extraordinarily high baseline NSIs rate (72.5 per 100 person-years) and the substantial IRR observed in the Infusion Center inevitably reflect a confounding of environmental risk with procedural volume. Each nurse in this setting performs significantly more percutaneous procedures per shift than their counterparts in general wards. Consequently, the comparative risks reported herein represent the overall occupational burden of the clinical environment rather than the intrinsic risk of a single needle maneuver. Future prospective research should leverage electronic health records or smart-disposal tracking to calculate procedure-adjusted incidence rates, thereby decoupling the frequency of exposure from the true per-procedure hazard. A within-Infusion-Center sensitivity analysis using procedural workload as the exposure denominator shows that the per-HCW headline finding (69% P3 vs. P1 reduction in the Infusion Center) is conservative: the per-procedure-unit reduction was 87%, consistent with a 2.5-fold higher procedural workload per HCW in P3 against which the observed risk reduction held. The within-Infusion-Center per-procedure sensitivity analysis uses a workload-score metric maintained by the department for performance metric management, which is a weighted composite of seven activity types rather than a strict count of percutaneous procedures. Some component activities (e.g. ambulation rounds) do not directly involve needle handling. The corresponding IRRs should therefore be interpreted as NSI hazard per workload-score unit, with the score serving as a workload-weighted proxy for procedural exposure rather than a procedure count proper. Comparable workload data were not available for the 38 control departments, so the per-procedure analysis cannot extend to the cross-sectional or interaction contrasts.

Second, the digitalized microlearning curriculum was launched in November 2024, providing less than 2 months of exposure within the P3 observation window. It may not have contributed meaningfully to the P3 association observed here. We therefore treat microlearning as an exploratory, prospective component of the bundle, included on *a priori* theoretical grounds to support long-term reinforcement and not as a demonstrated driver of the current effect. The P3 divergence, under the study's temporal logic, is most plausibly attributable to the leadership goal-setting (active from January 2023 onwards) and the dedicated preceptorship (active from H1 2023 onwards). An independent evaluation of microlearning requires a longitudinal follow-up beyond the current study window.

Third, generalizability of these findings beyond the present single tertiary hospital is limited. The intervention effect magnitude may differ in lower-throughput settings, in departments with different staffing models, and in hospitals with different baseline safety culture. External validity requires replication across hospital tiers (county-level, secondary, and academic) and across regions of China that differ in reporting culture. Transferability of the leadership-accountability component may in particular depend on the availability of a departmental champion, which is itself a non-trivial precondition.

Other limitations include bundled interventions preventing component-level efficacy attribution and survey-based mechanistic insights vulnerable to recall bias. We also did not assess injury severity or seroconversion outcomes, while weighting injuries by exposure risk would provide a more nuanced safety metric. Future multi-center studies should employ factorial designs, prospective observation and procedure-level electronic health records integration. Longitudinal tracking beyond the current head nurse's tenure will reveal whether gains persist or require ongoing leadership championing.

In addition, formal fidelity and adherence measures for individual intervention components (preceptor contact hours per HCW, microlearning completion rates) were not captured prospectively and cannot be reconstructed from routine records, which precludes component-level attribution. The dynamic early warning system was also derived and applied within the same hospital and was not externally validated. Formal sensitivity and specificity cannot be computed because no independent ground-truth label of a “true alarm-worthy event” existed at the department-year level. Operational deployment therefore requires external validation in a multi-center dataset and calibration against a labelled surveillance benchmark. Annual staffing-headcount data were not available year-by-year across the study window and a time-invariant current-headcount offset was used, while institutional verification confirmed departmental staffing stability across the window, residual misspecification of the denominator cannot be excluded and may affect within-group longitudinal contrasts if true staffing trajectories departed from the stability assumption.

## 5 Conclusion

This single-center, 7-year longitudinal observational study is consistent with the hypothesis that systematic behavior-based interventions are associated with sustained NSIs reduction in a high-risk setting despite healthcare system volatility. The Infusion Center's 66% risk reduction was maintained throughout P3 while the control departments rebounded to near pre-pandemic levels, a pattern consistent with the interpretation that leadership accountability, dedicated competency development and continuous behavioral reinforcement can buffer against post-pandemic environmental pressures. Rather than tolerating elevated NSIs rates in procedurally intensive departments, our findings suggest that healthcare systems may achieve more durable occupational-injury prevention by targeting evidence-based resources to empirically identified high-risk departments and by embedding protective behaviors into operational rituals through visible leadership accountability, setting-specific skill development and real-time feedback rather than relying on cognitive-safety training and generic knowledge dissemination alone.

## Data Availability

The original contributions presented in the study are included in the article/Supplementary material, further inquiries can be directed to the corresponding authors.

## References

[1] Bouya S Balouchi A Rafiemanesh H Amirshahi M Dastres M Moghadam MP . Global prevalence and device related causes of needle stick injuries among health care workers: a systematic review and meta-analysis. Ann Glob Health. (2020) 86:35. doi: 10.5334/aogh.2698 32346521 PMC7181946

[2] Wang T Liang Y Wu X Hao M A. large-scale survey on epidemiology and underreporting of needlestick and sharp injuries among healthcare workers in China. Front Public Health. (2023) 11:1292906. doi: 10.3389/fpubh.2023.1292906 38026416 PMC10652868

[3] Mengistu DA Tolera ST Demmu YM. Worldwide prevalence of occupational exposure to needle stick injury among healthcare workers: a systematic review and meta-analysis. Can J Infect Dis Med Microbiol. (2021) 2021:9019534. doi: 10.1155/2021/9019534 33564345 PMC7864758

[4] Cooke CE Stephens JM. Clinical, economic, and humanistic burden of needlestick injuries in healthcare workers. Med Devices. (2017) 10:225–35. doi: 10.2147/MDER.S140846 29033615 PMC5628664

[5] Association of Occupational Health Professionals in Healthcare (AOHP). AOHP Sharp Safety Exposure Report in Hospitals 2023. (2025). Available online at: https://www.aohp.org/aohp/portals/0/ (accessed March 10, 2026).

[6] Armenteros-Yeguas V Gárate-Echenique L Tomás-López MA Cristóbal-Domínguez E. Moreno-de Gusmão B, Miranda-Serrano E, et al. Prevalence of difficult venous access and associated risk factors in highly complex hospitalised patients. J Clin Nurs. (2017) 26:4267–75. doi: 10.1111/jocn.13750 28165645 PMC6084302

[7] Santos-Costa P Paiva-Santos F Bernardes RA Sousa LB Ventura F Faria J . “Difficult Intravenous Access in Older Adults with Cancer: Can Vein-Locating Technology Be Key for Vessel Health?”. In:Moguel E de Pinho LG Fonseca C, editor. Gerontechnology V. Cham, canton of Zug: Springer (2023). p. 241–50.

[8] Stojic J Grabovac V Lucijanic M. Needlestick and sharp injuries among healthcare workers prior to and during the coronavirus disease 2019 (COVID-19) pandemic. Infect Control Hosp Epidemiol. (2022) 43:1966–8. doi: 10.1017/ice.2021.498 34895375 PMC8692850

[9] Diktas H Oncul A Tahtasakal CA Sevgi DY Kaya O Cimenci N . What were the changes during the COVID-19 pandemic era concerning occupational risks among health care workers? J Infect Public Health. (2021) 14:1334–9. doi: 10.1016/j.jiph.2021.06.006 34172412 PMC8490998

[10] De Gagne JC Park HK Hall K Woodward A Yamane S Kim SS. Microlearning in health professions education: scoping review. JMIR Med Educ. (2019) 5:e13997. doi: 10.2196/13997 31339105 PMC6683654

[11] Zarshenas L Mehrabi M Karamdar L Keshavarzi MH Keshtkaran Z. The effect of micro-learning on learning and self-efficacy of nursing students: an interventional study. BMC Med Educ. (2022) 22:664. doi: 10.1186/s12909-022-03726-8 36071456 PMC9450813

[12] Des Jarlais DC Lyles C Crepaz N. TREND Group. Improving the reporting quality of nonrandomized evaluations of behavioral and public health interventions: the TREND statement. Am J Public Health. (2004) 94:361–6. doi: 10.2105/AJPH.94.3.361 14998794 PMC1448256

[13] Hoffmann TC Glasziou PP Boutron I Milne R Perera R Moher D . Better reporting of interventions: template for intervention description and replication (TIDieR) checklist and guide. BMJ. (2014) 348:g1687. doi: 10.1136/bmj.g1687 24609605

[14] Mubarak S Al Ghawrie H Ammar K Abuwardeh R. Needlestick and sharps injuries among healthcare workers in an oncology setting: a retrospective 7-year cross-sectional study. J Int Med Res. (2023) 51:3000605231206304. doi: 10.1177/03000605231206304 37871623 PMC10683567

[15] Zohar D Luria G A. multilevel model of safety climate: cross-level relationships between organization and group-level climates. J Appl Psychol. (2005) 90:616–28. doi: 10.1037/0021-9010.90.4.616 16060782

[16] Sexton JB Helmreich RL Neilands TB Rowan K Vella K Boyden J . The safety attitudes questionnaire: psychometric properties, benchmarking data, and emerging research. BMC Health Serv Res. (2006) 6:44. doi: 10.1186/1472-6963-6-44 16584553 PMC1481614

[17] McGaghie WC Issenberg SB Cohen ER Barsuk JH Wayne DB. Does simulation-based medical education with deliberate practice yield better results than traditional clinical education? a meta-analytic comparative review. Acad Med. (2011) 86:706–11. doi: 10.1097/ACM.0b013e318217e119 21512370 PMC3102783

[18] Woods D Dekker S Cook R Johannesen L Sarter N. Behind Human Error (2nd edition). London: Ashgate Publishing (2010). 271 p.

[19] Gawande A. The Checklist Manifesto: How to Get Things Right. New York: Metropolitan Books (2009). 224 p.

[20] Pronovost P Needham D Berenholtz S Sinopoli D Chu H Cosgrove S . An intervention to decrease catheter-related bloodstream infections in the ICU. N Engl J Med. (2006) 355:2725–32. doi: 10.1056/NEJMoa061115 17192537

[21] Tarigan LH Cifuentes M Quinn M Kriebel D. Prevention of needle-stick injuries in healthcare facilities: a meta-analysis. Infect Control Hosp Epidemiol. (2015) 36:823–9. doi: 10.1017/ice.2015.50 25765502

[22] Benneyan JC Lloyd RC Plsek PE. Statistical process control as a tool for research and healthcare improvement. Qual Saf Health Care. (2003) 12:458–64. doi: 10.1136/qhc.12.6.458 14645763 PMC1758030

[23] Barnett AG van der Pols JC Dobson AJ. Regression to the mean: what it is and how to deal with it. Int J Epidemiol. (2005) 34:215–20. doi: 10.1093/ije/dyh299 15333621

