# Supplementary material for: Disease burden, influencing factors and trend-based surveillance of needlestick injuries among healthcare workers: a seven-year longitudinal study and intervention evaluation at a tertiary hospital
Source: Front Public Health. 2026 Jun 11;14:1833892. doi: 10.3389/fpubh.2026.1833892 (PMC13294093; doi:10.3389/fpubh.2026.1833892)
Supplement: Supplementary file 1 [file Table_1.docx]

**Supplementary Material - Supplementary Table S1**

**TABLE S1. TIDieR-Based Description of the Safety Intervention Bundle**

| **TIDieR Item** | **1. Administrative Leadership ("Zero-NSI" Performance Metric)** | **2. Dedicated Preceptorship** | **3. Cloud-Based Video Microlearning Curriculum** |
| --- | --- | --- | --- |
| **WHAT — Intervention Description** | | | |
| **1. Brief Name** | | | |
| **Short descriptive name** | Zero-NSI leadership metric | Dedicated preceptorship | Digitalized microlearning curriculum |
| **2. Why — Theoretical Basis / Goal** | | | |
| **Underpinning theory or rationale** | Accountability theory and high-reliability organization principles: embedding NSIs elimination as a measurable performance indicator for department leadership creates normative pressure and aligns management incentives with front-line safety behavior. | Simulation-based medical education theory: expert-to-novice bedside coaching with deliberate practice reinforces correct sharps-handling technique in the authentic work context, allowing immediate corrective feedback. | Microlearning theory: short, modular, self-paced digital modules delivered on familiar mobile/web platforms overcome scheduling barriers and support spaced repetition of safety knowledge. |
| **3. What — Materials** | | | |
| **Physical or informational materials used** | Key performance indicator (KPI) dashboard visible to hospital administration and head-nurses; periodic performance review meetings; formal inclusion of NSIs rate in annual appraisal documentation. | Standardized competency checklist for safe sharps handling; observation logs completed by preceptors; department-level incident tracking sheet used during supervision sessions. | Eight-module cloud-based video curriculum depicting eight error-prone scenarios (needle hand-off, needle aiming at sharps container, infusion dressing left adhered to glove, hand-off while needle stuck in sharps container, needle bobbing while infusion dressing adhered to glove, needle pointing forward, incorrect needle withdrawal, and reaching into sharps container while handling needle); clinical skill evaluation in needle-related procedures after learning for each nurse. |
| **4. What — Procedures** | | | |
| **Activities and processes** | Head-nurse role redesigned to include explicit accountability for department-level NSIs rate. Target: zero NSI per period. Rate reported monthly by infection-control team to department head and hospital leadership. Non-attainment triggers structured review. | Newly hired or rotating nurses paired with trained preceptor nurse. Bedside coaching sessions conducted during live clinical shifts. Preceptor observes, models and corrects sharps-handling in real time. Expanded from pilot scheme to full departmental coverage through H1 2023. | All nurses assigned individual login to cloud platform. Modules completed at own pace during the initial phase of departmental orientation (first week) for newly hired or rotating nurses. Completion status recorded by platform. |
| **WHO — Providers and Recipients** | | | |
| **5. Who Provided — Interventionists** | | | |
| **Deliverers and their expertise** | Department head and hospital administration (strategic oversight); infection-control team (data reporting); head-nurse (operational accountability). | Designated preceptors — experienced senior nurses selected for clinical expertise and communication skills; trained in structured peer-coaching methods prior to preceptorship. | Self-directed by individual nurses via the cloud platform. Platform-side support (technical helpdesk) available. No facilitator required for module delivery. |
| **6. Who Received — Target Population** | | | |
| **Recipients and eligibility** | Head-nurse of the department (indirect exposure for all nurses via altered leadership behavior and safety climate). | Newly hired or rotating nurses in the department, prioritizing new starters and staff identified as high-risk on competency review. | All nurses in the department, irrespective of seniority or prior training history; completion mandatory for newly hired or rotating nurses per department policy from November 2024. |
| **HOW / WHERE — Delivery Mode and Setting** | | | |
| **7. How Delivered — Mode and Format** | | | |
| **Individual, group, or system-level; face-to-face vs. remote** | System-level / organizational. No direct face-to-face component with front-line nurses; change is mediated through leadership behavior and managerial climate. | Individual and small-group (1:1 or 1:2–3). Face-to-face, in the clinical environment during active patient-care shifts. | Individual, self-paced, fully remote / digital. Accessible via smartphone, tablet, or desktop browser; no synchronous component. |
| **8. Where — Setting** | | | |
| **Location of delivery** | Hospital administrative and management structures; department-level daily operations shaped by head-nurse accountability. | Clinical settings within the department (bedside/medication-preparation rooms /medical waste disposal rooms). | Any location with internet access (hospital, home, commute); no fixed venue required. |
| **WHEN / HOW MUCH — Timing, Dose, and Intensity** | | | |
| **9. When — Start Date and Phase** | | | |
| **Start date; study phase during which component was active** | January 2023. Active throughout the entire P3 observation window. | January 2023 (pilot phase H1 2023; full roll-out from Q3 2023 onwards). Active for the majority of the P3 window. | November 2024. Active for approximately 2 months of the P3 window (i.e., exposure was minimal within the study period). |
| **10. Intensity and Frequency** | | | |
| **Dose, duration, and frequency per recipient** | Continuous structural exposure (metric in place 24/7 as ambient accountability pressure). Monthly data review meetings (about 1 hour/month for head-nurse). Annual appraisal documentation. | Frequency not formally prescribed in pilot phase; targeted minimum of 2–5 observed sessions per nurse per month during roll-out. Each session 5–10 minutes embedded in a clinical shift. | 8 modules × 1–2 minutes each = 8–16 minutes total curriculum per nurse. Mandatory completion timeline for newly hired or rotating nurses: by end of the first week in the department. |
| **TAILORING — Adaptations and Modifications** | | | |
| **11. Tailoring** | | | |
| **Whether and how the intervention was tailored to individuals or subgroups** | Not tailored to individual nurses; uniform accountability standard applied to all head-nurses. | Preceptor matching based on department assignment and clinical specialty. Coaching content prioritized according to individual competency-check findings (e.g., more frequent sessions for nurses with identified high-risk practices). | Module sequence fixed; no adaptive branching. Platform allowed flexible pacing to accommodate shift patterns. Language: single language version at launch. |
| **12. Modifications During Delivery** | | | |
| **Changes made to the intervention after initial implementation** | None formally documented. | Scope expanded from pilot scheme (H1 2023) to full departmental coverage (Q3 2023); preceptor pool enlarged accordingly. | No modifications within the study window (launched November 2024; study period closes December 2024). |
| **HOW WELL — Fidelity and Uptake** | | | |
| **13. Fidelity — Planned Assessment** | | | |
| **Whether and how fidelity or adherence was to be monitored** | NSIs rate itself served as a de-facto outcome metric for the leadership target. No direct measure of head-nurse behavior fidelity was planned prospectively. | Preceptor contact-hours per nurse were not captured prospectively. Informal observation logs were kept but not systematically aggregated. | Platform-level completion tracking (module completion rates per nurse) was technically available via administrator dashboard but not extracted as part of the study data-collection protocol. |
| **14. Fidelity — Actual Assessment and Limitations** | | | |
| **Actual fidelity data obtained; candid acknowledgement of gaps** | Not formally measured. Head-nurse turnover during the period was low (1 replacement), limiting major accountability discontinuity, but engagement quality with the metric cannot be quantified. | Formal preceptor contact-hours per nurse not reconstructible from routine records with sufficient granularity. This precludes component-level attribution of effect. | Aggregate module completion rates were not extracted within the study period. Individual-level completion data are unavailable for analysis. Noted as a limitation in the manuscript. |

NSIs: needlestick injuries; P3: Phase 3 (post-COVID-19 pandemic recovery phase, January 2023–December 2024); Q: quarter; H: half.
